# Metformin promotes PEN2 expression to attenuate microglia-mediated neurotoxicity induced by HIV-1 Tat

**DOI:** 10.1007/s13365-025-01263-w

**Published:** 2025-06-04

**Authors:** Ya Shen, Tianli Xu, Yezi Sun, Kelun Zhang, Xiaojun Cao, Limin Shen, Mengjie Tang

**Affiliations:** 1 Department of Endocrinology, The Affiliated Zhangjiagang Hospital of Soochow University, Suzhou, 215123 Jiangsu China; 2 Department of Orthopedics, The Affiliated Zhangjiagang Hospital of Soochow University, Suzhou, 215123 Jiangsu China

**Keywords:** HAND Tat, Metformin, PEN2, Neurotoxicity

## Abstract

Metformin, a first-line drug used to treat type 2 diabetes mellitus (T2DM), also reduces neuroinflammation and improves motor and cognitive outcomes. Metformin binds to presenilin enhancer 2 (PEN2) and further enhances its therapeutic benefits. The mechanisms of HIV-associated neurocognitive disorders (HANDs) remain unclear. HIV-1 trans-activator of transcription (Tat) contributes to neurotoxicity in HAND. We revealed that PEN2 expression decreased markedly in HAND patients and Tat-infected microglia. Metformin (200 µM) treatment significantly reduced Tat-induced decreases in cell viability, oxidative stress, the proinflammatory response and excessive glutamate and iNOS release and had neuroprotective effects. Tat subsequently increased NF-κB activity, which was prominently suppressed during treatment. In addition, PEN2 knockdown in microglia dramatically reversed the neuroprotective effect of metformin against Tat. Our findings indicate that metformin binds PEN2 and modulates microglia-mediated HIV-1 Tat neurotoxicity in HAND.

## Introduction

Human immunodeficiency virus-1 (HIV-1) infection is a pandemic and is the major pathogen of acquired immunodeficiency syndrome (AIDS), infecting approximately 39 million people worldwide and continuously impacting 1–2 million new infections each year (Delpech 2022). Although combined antiretroviral therapy (cART) has been widely used, HIV-associated neurocognitive disorder (HAND) remains a significant challenge, afflicting 19–69% of the HIV-1-infected population (Wei et al. 2020). The main clinical manifestations of HAND are disturbances in cognitive, behavioural, motor, and autonomous functions, which are divided into the categories of asymptomatic neurocognitive impairment (ANI), mild neurocognitive disorder (MND), and HIV-associated dementia (HAD) (Rojas-Celis et al. 2019). All of these symptoms reduce the quality of life of infected individuals.

HIV enters the central nervous system (CNS) within 15 days of exposure (Spudich and Gonzalez-Scarano 2012). The mechanism by which HIV enters the immune-privileged brain is unknown. The “Trojan horse” model reveals that monocytes infected with the virus can cross the blood‒brain barrier (BBB) and secrete HIV into CNS-resident tissue macrophages and microglia (Bertrand et al. 2019; Gumbs et al. 2022), playing an important role in HAND pathogenesis (Borrajo et al. 2021; Ru et al. 2019). HIV infection of microglia results from defective mitophagy, reactive oxygen species (ROS), reactive nitrogen species (RNS) and persistent neuroinflammation via the production of interleukin-1 (IL-1), interleukin-6 (IL-6), tumour necrosis factor alpha (TNF-α), and C–C motif ligand 2 (CCL2), leading to neurodegenerative effects in HAND patients (Chivero et al. 2017; Mamik et al. 2017; Muzio et al. 2021).

HIV-1-encoded trans-activator of transcription (Tat), an 86–101 amino acid protein, is known to mediate viral replication (Marino et al. 2020). HIV-1 Tat promotes the transcription of long viral transcripts for the integrated provirus (Mamik and Ghorpade 2016). HIV-1 Tat is released mainly by reservoirs, including microglia, and then interacts with nearby cells in the CNS (Mele et al. 2018). HIV-1 Tat damages the BBB by downregulating tight junction proteins (Thompson et al. 2024). Treatment of human microglia with HIV-1 Tat promotes ROS, advanced glycation end products (AGEs), amyloid beta (Aβ) and proinflammatory mediators such as TNF-α, CCL2, IL-6, and CXCL10 in the brain (Chen et al. 2016; Jiang et al. 2017; Zhong et al. 2008). Astrocytes treated with HIV-1 Tat exhibit inducible nitric oxide synthase through the nuclear factor-κB (NF-κB) signalling pathway (Periyasamy et al. 2019). HIV-1 Tat promotes CD40 in microglia via the activation of NF-κB and the release of TNF-α, amplifying inflammation (Thompson et al. 2024). Inhibiting NF-κB signalling might be an effective strategy to repress Tat-induced neurodegeneration in microglia.

Metformin, the usual first-line drug used to treat type 2 diabetes mellitus (T2DM), has other beneficial effects, such as anticancer effects, reduced body weight, and increased hepatic fat content (Barzilai et al. 2016; Foretz et al. 2019; Galal et al. 2024; Rena et al. 2017). Metformin has also been reported to activate the adenosine monophosphate-activated protein kinase (AMPK) pathway to inhibit microglial cell activation and polarization, reduce neuroinflammation, and improve motor and cognitive outcomes (Kodali et al. 2021; Ruddy et al. 2019; Wang et al. 2021). Teng Ma et al. revealed that metformin binds to presenilin enhancer 2 (PEN2), a regulatory component of the gamma-secretase complex, initiates v-ATPase inhibition via ATP6 AP1 and further regulates the activation of MAPK. In vivo, PEN2 knockout abolishes the metformin-induced reduction in hepatic fat content, attenuates glucose-induced effects and antiaging (Ma et al. 2022). However, whether metformin regulates microglia-mediated HIV-1 Tat neurotoxicity by binding to PEN2 remains to be investigated.

In the present study, we found that the expression of PEN2 decreased in HAND patients. Furthermore, metformin alleviated NF-κB signalling, ROS, AGEs, proinflammatory mediators and neuronal apoptosis in HIV-1 Tat-infected BV-2 microglia by binding to PEN2. These data confirmed that metformin promotes PEN2 expression to exert its anti-inflammatory and neuroprotective effects.

## Materials and methods

### GEO dataset

We obtained the gene expression dataset GSE28160 (Borjabad et al. 2011) from the Gene Expression Omnibus (GEO, http://www.ncbi.nlm.nih.gov/geo/) database. The GSE28160 dataset included brain tissue samples from seven treated and eight untreated HAND patients and six uninfected controls. Studies have used microarray analysis of postmortem brain tissues to determine the effectiveness of ART in the brain and to identify the molecular signatures of HAND under ART. The expression of PEN2 in HAND patients and uninfected controls was analysed with GraphPad Prism 8.3.0 software. The data were downloaded from the GEO database, and approval from the ethics committee was not needed.

### Cell culture

Mouse BV-2 microglia and HT-22 neuronal cells were purchased from the Cell Bank of the Chinese Academy of Sciences (Shanghai, China). The cells were preserved in DMEM (Gibco) supplemented with 10% foetal bovine serum (Lonicera, A511-001, USA), 100 UI/ml penicillin and 100 mg/ml streptomycin and cultured in a warm, moist environment (37 °C, 5% CO_2_). BV-2 microglia were exposed to metformin at the indicated concentrations.

### Cell transduction

The cells were transduced with short hairpin RNA or flag tagged-Tat (86 aa) mRNA via lentiviral vectors. Oligobio Co., Ltd. (Beijing, China, viral titers = 1*10^8^) devised and packaged the lentiviral vectors. The BV-2 cells were infected with lentivirus in culture medium to reach an MOI (multiplicity of infection) of 80. After 48 h of infection, GFP (green fluorescent protein)-positive cells were overexpressed and purified by adding hygromycin B (50 μg/ml) to the culture medium. Finally, Western blotting was used to determine the efficiency of infection efficiency. The lentiviruses with empty vector and Tat were termed LV-Vector and LV-Tat, respectively. The lentiviruses with scrambled shRNA or PEN2 shRNA are represented as shScramble and shPEN2, respectively.

The shRNA sequence used was 5′-GCT ACA CTA TCG AGC AAT T-3′ for shScramble. 5′-GCC AAA TCA AAG GCT ATG TTT-3′ for shPEN2.

### RNA extraction and polymerase chain reaction (PCR)

Total RNA from cultured cells was extracted with TRIzol reagent (Invitrogen). Following reverse transcription (TaKaRa, Japan), 2 × QuantiNova SYBR Green PCR Master Mix (QIAGEN) was used to measure the mRNA levels via a T100™ Thermal Cycler (Bio‐Rad). The PCR products were separated on 1% agarose gels. Following agarose gel electrophoresis (AGE), the intensity of each band was quantified with ImageJ software. The mRNA levels were normalized to those of β-actin. The sequences of primers used were as follows: 5′‐ATCTG-GCACCACACCTTCTACAAT‐3′ and 5′‐CACGCTCGGTCAGGATCTTCAT‐3′ for β-actin and 5′‐GCGGGTATCCAATGAGGAGAAGT‐3′ and 5′‐GTGATCCAGGTGGCGAGAATGA‐3′ for PEN2.

### Protein extraction and Western blot analysis

To obtain total cellular protein, microglia were lysed in RIPA buffer (Beyotime) containing 0.5 mM PMSF (Sigma) and 1 × phosphatase inhibitor (Roche). Protein samples were subjected to sodium dodecyl sulfate–polyacrylamide gel electrophoresis and then transferred to polyvinylidene fluoride (PVDF) membranes (Bio-Rad). The PVDF membranes were incubated at room temperature for 2 h in 5% fat-free milk and then incubated with primary antibodies at 4 °C overnight. After another hour of incubation with the PVDF membranes and secondary antibodies, the membranes were incubated with an enhanced chemiluminescence detection kit (Proteintech). The grayscale levels were measured with ImageJ software. The protein levels were normalized to grayscale values of β-actin. The following antibodies were used: anti-β-actin (Proteintech, 66,009–1-Ig, 1:5000), anti-FLAG tag (Cell Signaling Technology, #14,793, 1:1000), anti-PEN2 (Cell Signaling Technology, #8598, 1:1000), anti-NF-κB p65 (Wanleibio, WL01980, 1:500), anti-phosphorylated NF-κB p65 (P-NF-κB p65) (Wanleibio, WL02169, 1:500), EAAT2 (Cell Signaling Technology, #3838, 1:1000), iNOS (Cell Signaling Technology, #2928, 1:1000), IBA1 (Proteintech, MA5–27726, 1:1000), HRP-conjugated goat anti-mouse IgG (Jackson, 115–035–003, 1:10,000), and HRP-conjugated goat anti-rabbit IgG (Jackson, 111–035–003, 1:10,000) antibodies.

### Enzyme‑linked immunosorbent assay

Enzyme-linked immunosorbent assay (ELISA) was used to identify inflammatory cytokines in the cell culture supernatants following the manufacturer’s protocol. The ELISA kits used were as follows: a mouse IL-6 beta sandwich ELISA kit (Proteintech, KE10003) and a mouse TNF-α ELISA kit (Proteintech, KE10002), Mouse MCP-1 ELISA Kit (Proteintech, KE10006). A mouse CXCL10 ELISA Kit (Thermo Fisher Scientific, BMS6018) was used.

### Immunofluorescence staining

Immunofluorescence staining was performed to measure IBA1 expression in microglia. Briefly, BV-2 microglia were seeded in 24-well plates, and the density of each well was 2 × 10^4^ cells. After being washed twice with PBS, the cells were fixed in 4% paraformaldehyde. Following permeabilization and blocking, the cells were continuously incubated with primary antibodies and secondary antibodies. Antifade mounting medium with DAPI (Beyotime) was used for mounting. The cells were inspected and recorded in ten random visual fields per well under a microscope at 200 × magnification. An anti-Iba1 antibody (Proteintech, 10,904–1-AP, 1:100) was used. ImageJ software was used to measure the expression of IBA1.

### Superoxide dismutase (SOD), malondialdehyde (MDA) measurement

The level of MDA in microglia was measured with a Lipid Peroxidation MDA Assay Kit (Beyotime, S0131M), and the absorbance was measured at 532 nm by using a Synergy H1 Microplate Reader (Bio-Tek). The activity of SOD in microglia was detected with a Total Superoxide Dismutase Assay Kit with WST-8 (Beyotime, S0101M), and the absorbance was detected at 450 nm by using a Synergy H1 Microplate Reader (Bio‐Tek).

### Reactive oxygen species (ROS) staining

Following the recommended instructions, the intracellular ROS dihydroethidium (DHE) assay (Beyotime, S0033M) and 2′,7′-dichlorofluorescin diacetate (DCF-DA) assay (Bestbio, BB-46,052) were performed. DHE was transformed to red fluorescent ethidium by superoxide and observed under a fluorescence microscope at 200 × magnification. DCF-DA was converted into green fluorescent DCF by intracellular ROS and was measured at 488 nm excitation and 525 nm emission by using a Synergy H1 Microplate Reader (Bio-Tek). The average absorbance of ten replicates was calculated.

### Lactate dehydrogenase (LDH) assay and Cell counting kit‑8 assay

Lactate dehydrogenase and cell counting kit-8 (CCK-8) assays were performed to assess cell viability. HT-22 neuronal cells were placed in 96-well plates at a density of 1 × 10^3^ cells per well. The HT-22 neuronal cells were washed twice with PBS before analysis and then supplied with CCK-8 reagents (Dojindo Laboratories, Kumamoto, Japan) following the recommended protocol. The absorbance was analysed at 450 nm through a Synergy H1 Microplate Reader (Bio-Tek). The average absorbance was calculated with six replicates. The levels of LDH release in the HT22 cell culture supernatants were measured via an LDH assay kit (Beyotime Biotechnology, Shanghai, China). In brief, NAD + was converted into NADH by LDH, leading to generate formazan. The calculation formula: %cytotoxicity = (LDH release-Blank control) (OD492)/(Maximum-Blank control)(OD492). The absorbance was measured at 490 nm via a Synergy H1 Microplate Reader (Bio-Tek). The average absorbance was calculated with eight replicates.

### Assessment of extracellular glutamate and nitric oxide levels

The levels of glutamate in BV-2 microglia were determined with a glutamate assay kit (Sigma‒Aldrich, MAK330). In brief, glutamate dehydrogenase catalyses the oxidation of glutamate, in which the formed NADH reduce formazan. The absorbance of the formazan was measured at 565 nm. The nitric oxide (NO) concentration in microglia was measured with an NO assay kit (Abcam, Ab272517) according to the manufacturer’s instructions.

### Statistical analyses

All quantifications were conducted in more than three independent experiments. Comparison of two groups was using an unpaired Student’s *t*-test or one-way analysis of variance (ANOVA) followed by the Bonferroni post hoc test. The measurement data are presented as the means ± standard deviations (SDs). *P* < 0.05 indicated statistical significance. Statistical analyses were performed with GraphPad Prism 8.3.0 software.

## Results

### Tat decreases PEN2 expression in HAND patients and BV-2 microglia

**Fig. 1:**
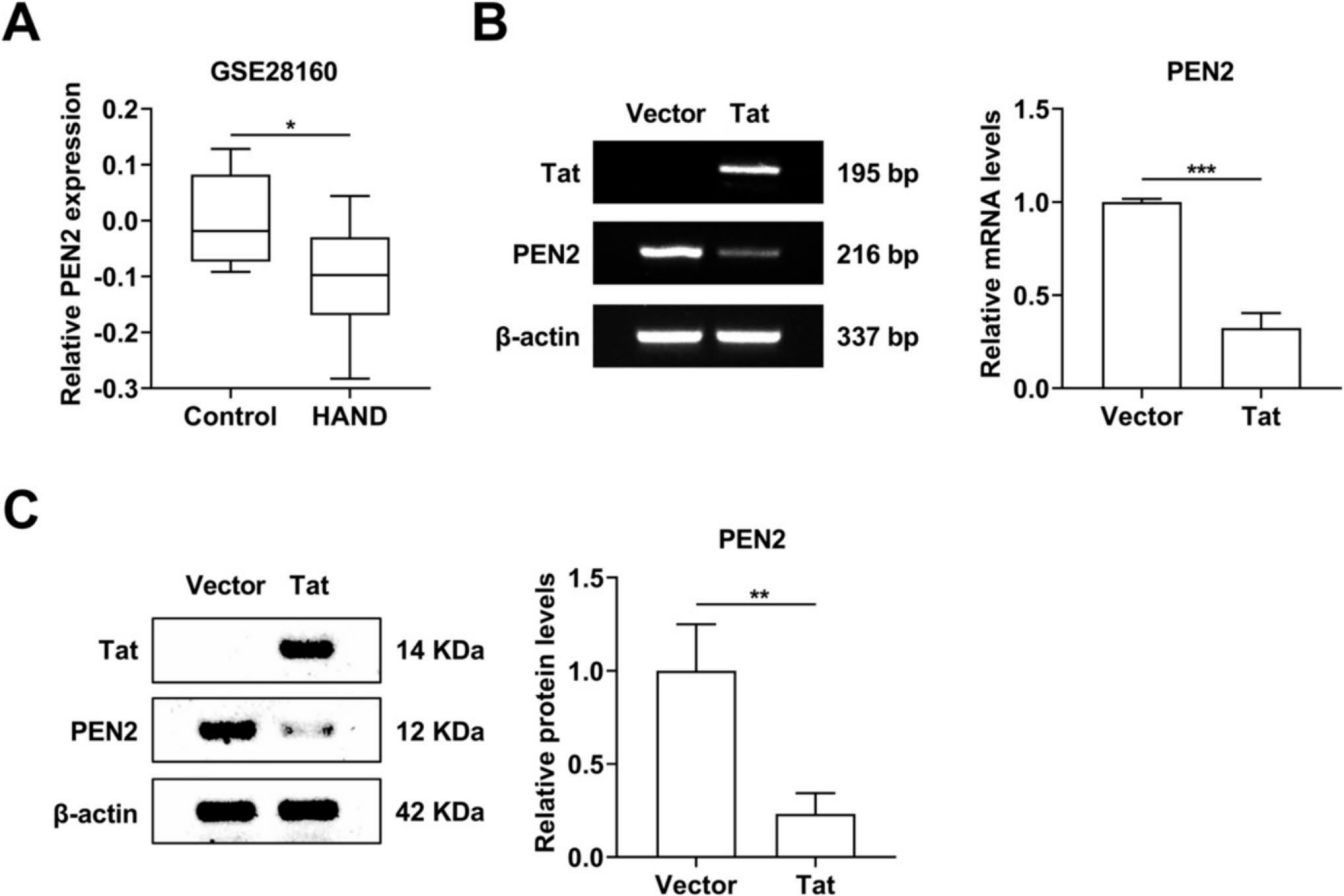
BV-2 microglial cells were transduced with LV-Vector or LV-Tat for 48 h. **A** Based on the gene expression dataset GSE28160, PEN2 expression decreased in HAND patients. **B** Agarose gel Electrophoresis analyzed Tat and PEN2 expression at mRNA levels. **C** Representative Western blot analysis of Tat and PEN2 expression at protein level. *n* = 3 for each group, * P < 0.05, **P < 0.01, ***P < 0.001, two-tailed* t*-test

On the basis of the data downloaded from the GEO database, we analysed the expression of PEN2 in HAND patients and normal controls and found that PEN2 expression decreased significantly in HAND patients (Fig. 1A). To test whether PEN2 is involved in Tat-induced microglial cell activation, agarose gel electrophoresis was performed to measure the expression of PEN2. As shown in Fig. 1B, the mRNA expression level of PEN2 decreased significantly after overexpression of Tat. A western blot analysis was performed to measure PEN2 protein expression, and the results indicated that Tat decreased PEN2 protein expression in BV-2 microglia (Fig. 1C). These results indicate that Tat downregulates PEN2 expression in BV-2 microglia.

### Metformin suppresses Tat-induced IBA1 protein expression in a dose-dependent manner

**Fig. 2:**
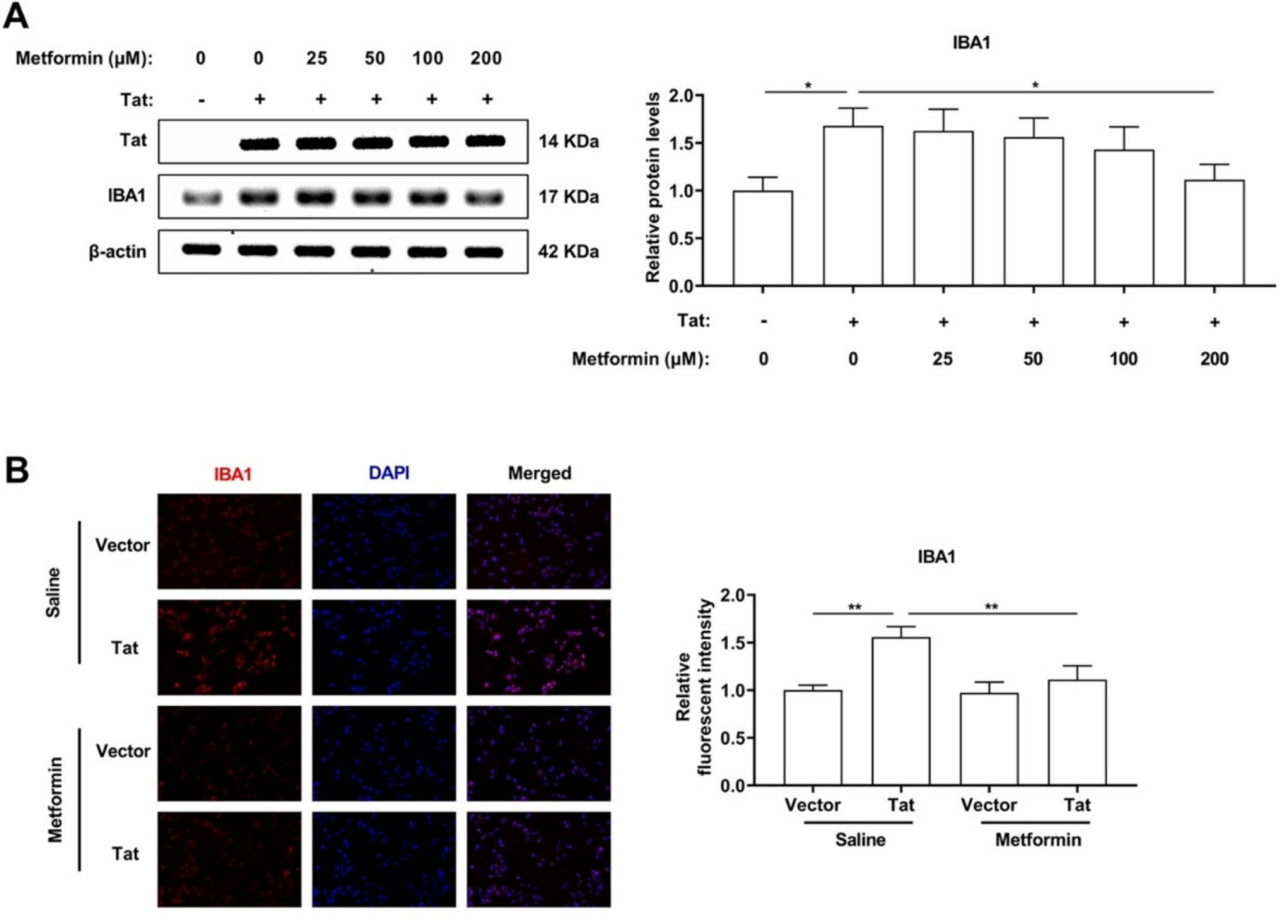
BV-2 microglial cells were transduced with LV-Tat for 48 h. **A** Representative Western blot analysis of Tat and IBA1 expression at protein levels and quantitative analysis of gray levels under different concentrations of metformin. **B** Immunofluorescence staining for IBA1 (red) and the histogram showed quantitative immunofluorescence analysis of IBA1 when metformin at concentrations of 200 µm. Scale bars, 50 μm. *n* = 3 for each group, *P < 0.05, **P < 0.01, two-tailed* t*-test

We first performed Western blotting (WB) to detect the effects of metformin on phenotype-specific markers of microglia (IBA1) and Tat. Metformin treatment caused a dose-dependent reduction in IBA1 protein expression. IBA1 protein expression decreased significantly when metformin was used at a concentration of 200 µm (Fig. 2A). In addition, immunofluorescence staining revealed a considerable reduction in Tat-induced IBA1 expression when metformin was used at a concentration of 200 µm (Fig. 2B). As a result, we performed the following experiments at a concentration of 200 µM metformin.

### Metformin reduces Tat-induced oxidative stress in BV-2 microglia cells by regulating PEN2 expression

**Fig. 3:**
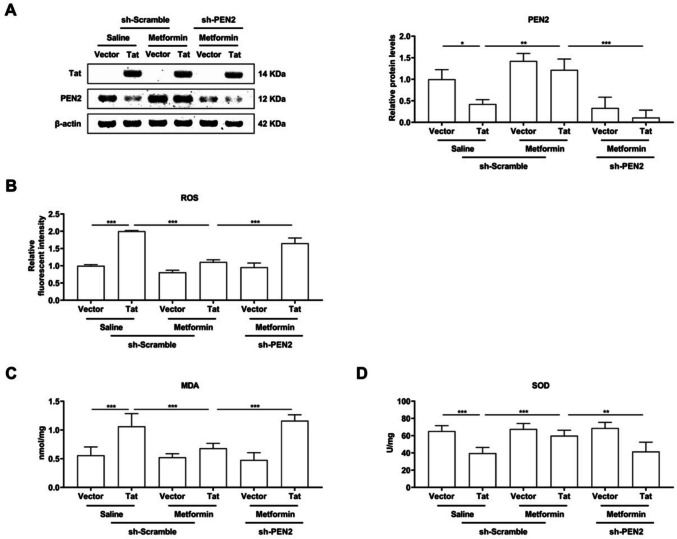
BV-2 microglial cells were transduced with LV-Vector or LV-flag-Tat for 48 h, treated with saline or metformin for 24 h, as well as LV-shScramble or LV-shPEN2. **A** Representative Western blot analysis of Tat and PEN2 expression at protein levels and quantitative analysis of gray levels, respectively. **B** DCFH-DA analysis of ROS. **C** Measurement of the Levels of MDA. **D** Determination of the activity of SOD. The results revealed that metformin reduces Tat-induced oxidative stress in BV-2 microglia cells by regulating PEN2 expression. *n* = 3 for each group, *P < 0.05, **P < 0.01, ***P < 0.001, two-tailed* t*-test

To understand the role of metformin and PEN2 in Tat-induced oxidative stress in BV-2 microglia, the following oxidative stresses, reactive oxygen species (ROS), malondialdehyde (MDA) and the key element involved in the defence against ROS, superoxide dismutase (SOD), were analysed. BV-2 microglia were first transduced with LV-Vector or LV-Flag-Tat for 48 h, followed by transduction with saline or metformin and LV-shScramble or LV-shPEN2 for another 24 h. Notably, PEN4 was reduced most significantly by sh-PEN2 (Fig. 3A). DCF-DA staining revealed that Tat significantly increased the levels of ROS and MDA but decreased the level of SOD in Tat-infected BV-2 microglia. Intriguingly, 200 µM metformin treatment clearly decreased the levels of ROS and MDA and increased the level of SOD in Tat-infected BV-2 microglia (Fig. 3B-D). The antioxidative effect of metformin on Tat-infected BV-2 microglia was reversed when PEN2 was knocked down (Fig. 3B-D).

### Metformin promotes PEN2 expression to suppress Tat-induced NF‑κB signalling activation and the inflammatory response in BV-2 microglia

**Fig. 4:**
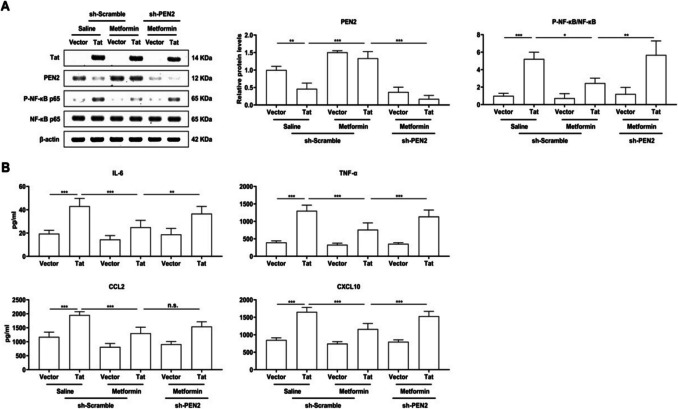
BV-2 microglial cells were transduced with LV-Vector or LV-flag-Tat for 48 h, treated with saline or metformin for 24 h, as well as LV-shScramble or LV-shPEN2. **A** Representative Western blot analysis of Tat, PEN2, P-NF-κB p65, and NF-κB p65 expression at protein levels and quantitative analysis of gray levels, respectively. **B** ELISA analysis of IL-6, TNF-α, CCL2 and CXCL10 in cell culture supernatants. The studies showed that metformin promotes PEN2 expression to suppress Tat-induced NF‑κB signalling activation and the inflammatory response in BV-2 microglia. *n* = 3 for each group, *P < 0.05, **P < 0.01, ***P < 0.001, two-tailed* t*-test

To investigate the role of PEN2 in Tat-induced microglial inflammation, we transduced LV-shRNA targeting PEN2 to silence it, and LV-shScramble was used as a control. WB revealed that PEN4 was reduced most significantly by sh-PEN2 (Fig. 4A). Multiple studies have shown that metformin inhibits NF-κB signalling and reduces the inflammatory response. Furthermore, to determine whether metformin mediates Tat-induced NF-κB signalling and inflammation, 200 µm metformin was applied to BV-2 microglia. Thus, BV-2 microglia were transduced with LV-Vector or LV-Flag-Tat for 48 h, followed by transduction with saline or metformin and LV-shScramble or LV-shPEN2 for another 24 h. Metformin significantly inhibited Tat-induced P-NF-κB p65 expression and NF-κB p65 expression at the protein level. Interestingly, this inhibition was reversed when PEN2 was knocked down (Fig. 4A). The proinflammatory cytokine and chemokine levels were also measured. As expected, metformin obviously decreased Tat-induced IL-6, TNF-α, CCL2 and CXCL10 expression, and this downregulation occurred when PEN2 was knocked down (Fig. 4B).

### Metformin inhibits glutamate neurotoxicity in Tat-transduced BV-2 microglia by targeting PEN2

**Fig. 5:**
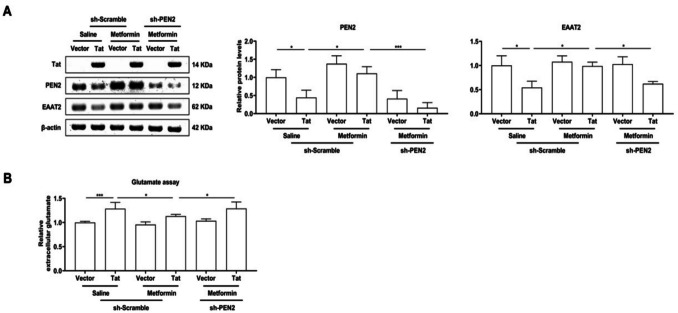
BV-2 microglial cells were transduced with LV-Vector or LV-flag-Tat for 48 h, treated with saline or metformin for 24 h, as well as LV-shScramble or LV-shPEN2. **A** Representative Western blot analysis of Tat, PEN2 and EAAT2 expression at protein levels and quantitative analysis of gray levels, respectively. **B** Measurement of extracellular glutamate levels. We detected that metformin inhibits glutamate neurotoxicity in Tat-transduced BV-2 microglia by targeting PEN2. *n* = 3 for each group, *P < 0.05, **P < 0.01, ***P < 0.001, two-tailed* t*-test

Previous research (Oruc et al. 2024) has indicated that metformin exerts neuroprotective effects during glutamate neurotoxicity. Considering that glutamate toxicity damages neurons, we tested excitatory amino acid transporter (EAAT) 2 expression and glutamate release in BV-2 microglia. Metformin treatment markedly inhibited glutamate release and promoted glutamate transport during Tat infection, which was significantly suppressed by PEN2 knockdown (Fig. 5A, B).

### Metformin reverses Tat-induced iNOS expression and NO production in BV-2 microglia

**Fig. 6:**
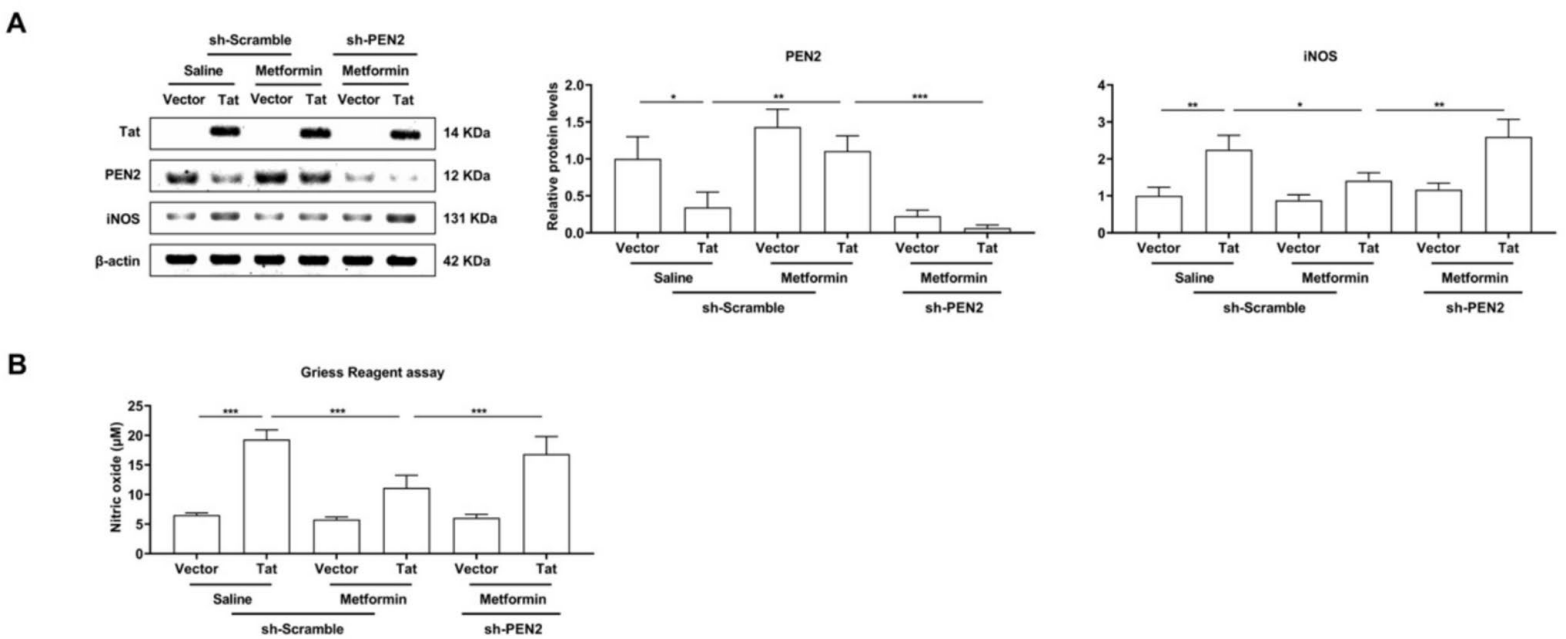
BV-2 microglial cells were transduced with LV-Vector or LV-flag-Tat for 48 h, treated with saline or metformin for 24 h, as well as LV-shScramble or LV-shPEN2. A. Representative Western blot analysis of Tat, PEN2 and iNOS expression at protein levels and quantitative analysis of gray levels, respectively. B. Measurement of nitric oxide levels. The metformin reverses Tat-induced iNOS expression and NO production in BV-2 microglia. *n* = 3 for each group, *P < 0.05, **P < 0.01, ***P < 0.001, two-tailed* t*-test

In addition to the inflammatory response and oxidative stress, type 2 nitric oxide synthase is subsequently activated in Tat-induced microglia (Mangino et al. 2015). Consistent with previous research (Peng et al. 2022), western blotting analysis revealed that metformin treatment markedly decreased inducible nitric oxide synthase (iNOS) expression and that knocking out PEN2 abolished the metformin-mediated reduction (Fig. 6A). A similar trend was detected in the production of nitric oxide (NO) (Fig. 6B).

### Metformin increases PEN2 expression to suppress Tat-induced neuronal apoptosis

**Fig. 7:**
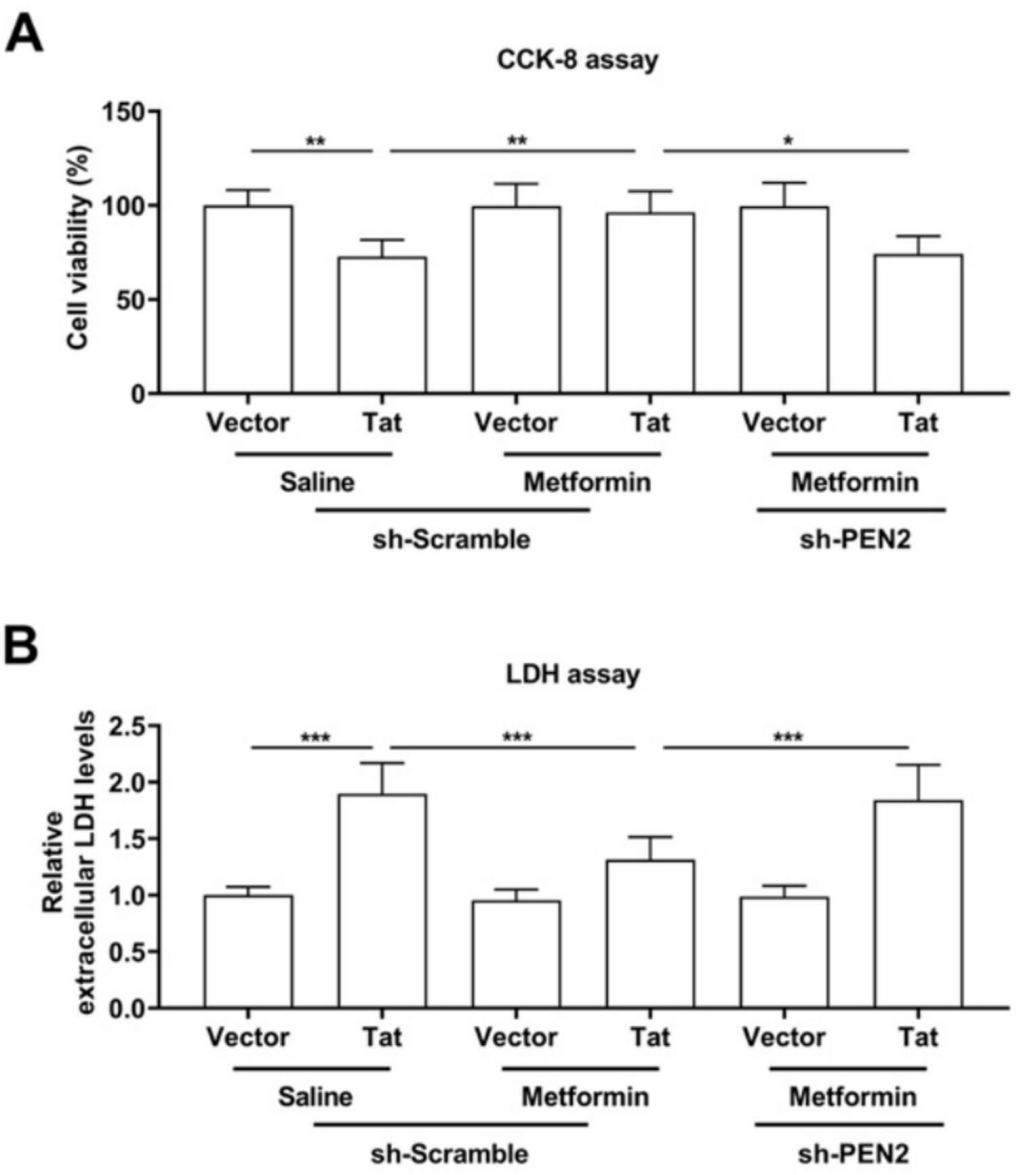
BV-2 microglial cells were transduced with LV-Vector or LV-flag-Tat for 48 h, treated with saline or metformin for 24 h, as well as LV-shScramble or LV-shPEN2. **A** Cell culture supernatants were collected as the MCM and added into HT-22 neuronal cells to incubate for another 24 h. CCK-8 analysis of cell viability. **B** Measurement of extracellular LDH levels. *n* = 3 for each group, *P < 0.05, **P < 0.01, ***P < 0.001, two-tailed* t*-test

To confirm the role of metformin and PEN2 expression in neuroprotective effects. Microglia-derived conditioned medium (MCM), which was collected from the culture supernatants of transduced BV-2 microglia, was added to HT-22 neuronal cells and incubated for at least 24 h. Cell viability was explored with a CCK-8 assay. HT-22 cell viability in response to the MCM derived from Tat-treated BV-2 microglia clearly increased after treatment with metformin and decreased when PEN2 was depleted (Fig. 7A). Moreover, the degree of cellular injury associated with LDH content in HT-22 cells was assessed. The LDH content increased significantly in HT-22 cells incubated with MCMs derived from Tat-treated BV-2 microglia, decreased when the cells were treated with metformin, and increased when PEN2 was depleted (Fig. 7B). Therefore, we believe that PEN2 knockdown decreased the neuroprotective effect of metformin in BV-2 microglia infected with Tat.

## Discussion

Although the introduction of cART has made HIV-1 infection a treatment condition, up to 50% of HIV-1-positive individuals continue to suffer from HAND (Heaton et al. 2010). The development and progression of HAND are complex and poorly understood. Tat, a key factor of HIV-1-encoded neurotoxic elements, contributes to a lasting low-level inflammatory response and neurodegeneration in microglia (Jadhav and Nema 2021; Williams et al. 2020). Previous studies have revealed that metformin plays a beneficial role in Alzheimer’s disease (Li et al. 2012) and spinal cord injury (Zhang et al. 2017) patients. Moreover, metformin regulates inflammation and displays neuroprotective effects by activating the AMPK signalling pathway and inhibiting NF-κB activity in microglia (Abdi et al. 2021). PEN2, a regulatory component of the gamma-secretase complex, binds to metformin and ensures that it has therapeutic benefits (Ma et al. 2022). However, whether metformin could play an important role in the evaluation of HAND remains unclear. The present study confirmed that PEN2 expression was markedly lower in HAND patients and that its expression was downregulated during Tat infection in BV-2 microglia. Moreover, we demonstrated for the first time that metformin treatment suppressed Tat-induced microglial oxidative stress, inhibited the NF-κB signalling pathway, attenuated the release of proinflammatory cytokines, and decreased the release of glutamate and iNOS in BV-2 microglia. We also observed that treatment with 200 µM metformin significantly restored microglial viability. Intriguingly, knocking out PEN2 abolished the beneficial effects of metformin in Tat-infected microglia, suggesting that metformin might alleviate the progression of microglia-mediated HIV-1 Tat neurotoxicity by targeting PEN2.

Previous studies have shown that patients with long-term suppression of HIV-1 virus expression exhibit activation of microglia on brain positron emission tomography (Garvey et al. 2014). Additionally, in HIV-1 Tat rats, the number (Repunte-Canonigo et al. 2014) and morphology (Rowson et al. 2016) of microglia are prominently altered. Various studies have reported that Tat-induced neurotoxicity consists of Tat itself and the glial cell-derived inflammatory response. Tat-infected microglia induced a proinflammatory phenotype and secreted interferon-gamma (IFN-γ) and TNF-α. The inflammatory cytokines IL-6, CCL2, CXCL10, glutamate, ROS and iNOS are then released, leading to neurotoxicity (Devanney et al. 2020). In accordance with previous reports, our data revealed that the levels of IL-6, TNF-α, CCL2, and CXCL10, as well as oxidative stress and the release of glutamate and iNOS, were significantly increased in Tat-infected BV-2 microglia. Metformin, a first-line therapy for T2DM treatment, also exerts anti-inflammatory effects on microglia and improves motor and cognitive outcomes (Kodali et al. 2021; Ruddy et al. 2019; Wang et al. 2021). Teng Ma et al. revealed that metformin binds PEN2 and exerts its therapeutic benefits (Ma et al. 2022). As expected, our results showed that 200 µM metformin treatment significantly reversed Tat-induced neurotoxicity and that knocking out PEN2 markedly abolished the metformin-mediated effects. Additionally, Tat promotes lysosome and neurotoxic microRNA (miRNA) exocytosis, resulting in microglial dysfunction (Ma et al. 2022). Whether metformin promotes PEN2 expression to reduce the release of lysosomal and neurotoxic miRNAs remains to be explored.

In addition to being used as a treatment for diabetes, metformin has also been reported to have anti-inflammatory, anti-weight, anticancer and antiaging effects (Barzilai et al. 2016; Foretz et al. 2019; Galal et al. 2024; Rena et al. 2017). Metformin administration significantly attenuated the inflammatory response and generated neuroprotective effects in microglia (Abdi et al. 2021). Metformin activates the AMPK pathway to exert protective effects and the AMPK pathway inhibits the inflammatory response by reducing NF-κB activity and further stimulates various downstream pathways (Abdi et al. 2021). HIV-1 Tat is vital in virus replication and stimulates the NF-κB signalling pathway to induce neuroinflammation in microglia (Mamik and Ghorpade 2016). However, the effect of metformin treatment on the activation of the NF-κB signalling pathway in Tat-infected microglia has not been explored. In the present study, we demonstrated that HIV-1 Tat can activate the NF-κB signalling pathway and subsequently regulate the secretion of cytokines. Surprisingly, metformin treatment clearly suppressed Tat-induced activation of the NF-κB signalling pathway, and depletion of PEN2 significantly reversed these effects. Therefore, metformin promotes PEN2 levels to downregulate NF-κB signalling pathway expression and further elaborates neuroprotective effects in Tat-transduced BV-2 microglia. While the metformin concentration used in this study (200 µM) exceeds typical clinical plasma levels (Foretz et al. 2019), this dose aligns with established in vitro models investigating PEN2-dependent lysosomal AMPK signaling in microglia (Labuzek et al. 2010). Future studies are needed to validate these findings in animal models or human microglial cultures using clinically relevant doses.

HIV-1 infection of microglia can alter cell activation, viability and metabolism (Stenovec et al. 2019). Recent research revealed that the inflammatory response in HIV-infected microglia generates a toxic environment and ultimately disturbs neuron function and survival (Kong et al. 2024). Consistent with these findings, we detected that microglial viability was markedly decreased during HIV-1 Tat infection. As expected, metformin treatment reversed the decrease in cell viability. We subsequently revealed that silencing PEN2 expression suppressed microglial viability again, suggesting that metformin promoted cell viability in Tat-transduced microglia by binding to PEN2.

Nevertheless, there are several limitations in the present study. First, considering the inadequate resources, animal experiments could not be conducted, the absence of in vivo validation in HAND-relevant models precludes assessment of systemic efficacy, off-target interactions, or blood–brain barrier penetration dynamics. This gap critically limits clinical translation. The dose-optimized studies in murine HAND models to bridge mechanistic findings with translational relevance should be conducted. Second, the molecular mechanism by which metformin targets PEN2 to attenuate neurotoxicity in Tat-infected microglia remains to be explored in further studies. Third, while metformin’s anti-inflammatory effects are often linked to AMPK activation, our findings highlight PEN2 as a critical mediator in mitigating HIV-Tat-induced microglial inflammation. Nevertheless, we acknowledge that metformin’s pleiotropic actions, including AMPK-independent effects, may contribute to its in vivo anti-inflammatory profile. Future studies using PEN2-deficient animal models or single-cell transcriptomics of patient-derived microglia will help delineate tissue-specific versus off-target mechanisms. Finally, while our analysis of the GSE28160 dataset revealed PEN2 downregulation in HAND patients, the reliance on a single cohort introduces potential selection bias and limits generalizability. The additional validation using independent datasets or patient-derived samples would strengthen our conclusions.

## Conclusion

**Fig. 8:**
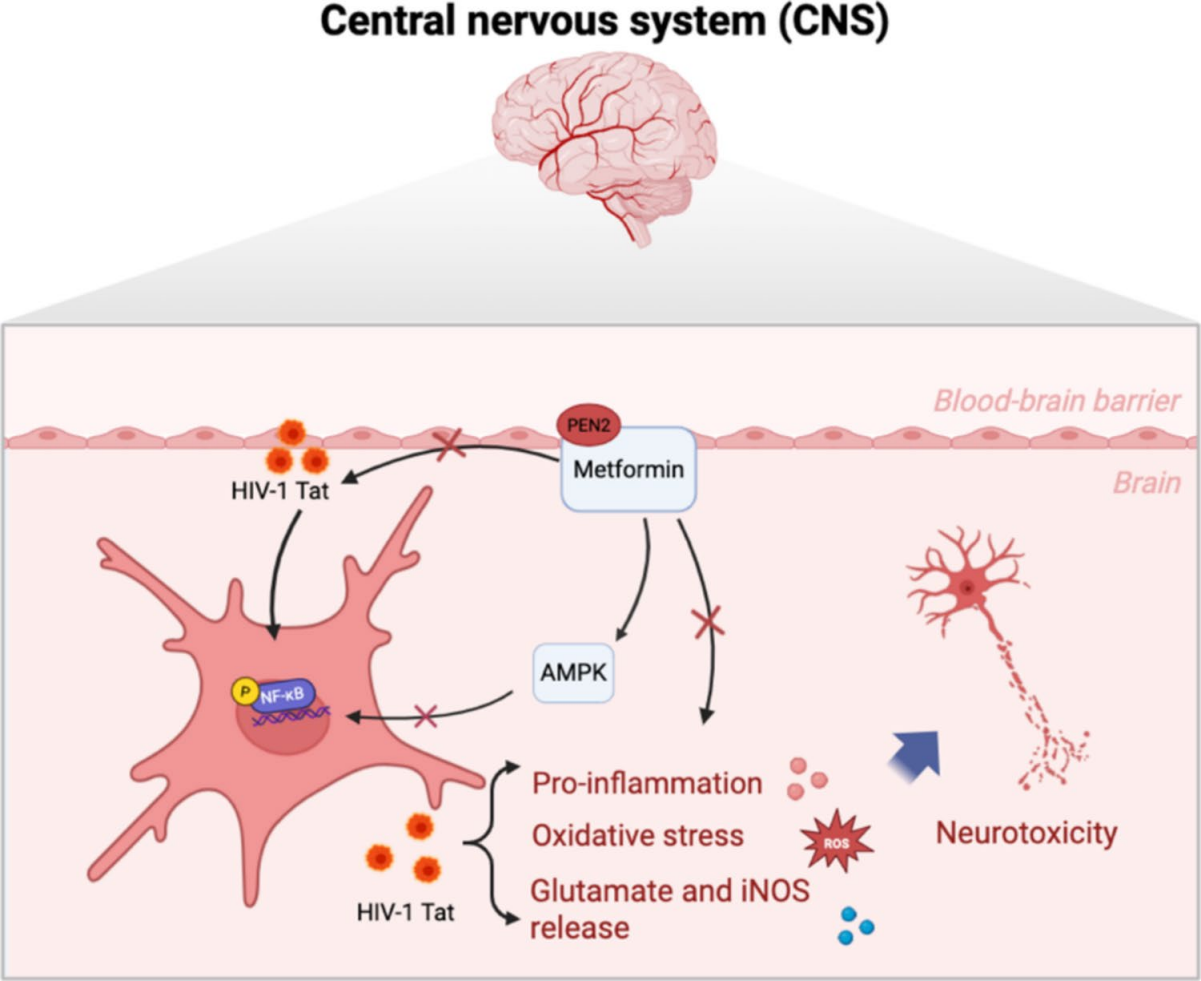
Schematic diagram of metformin promotes PEN2 expression to attenuate microglia-mediated HIV-1 Tat neurotoxicity. The metformin treatment attenuated neuroinflammatory, inhibited NF-κB activity, suppressed oxidative stress and promoted cell activation in Tat infected microglia cells via targeting to PEN2

In the present study, we demonstrated for the first time that metformin treatment attenuated neuroinflammation, inhibited NF-κB activity, suppressed oxidative stress and promoted cell activation in Tat-infected microglia by targeting PEN2 (Fig. 8). Our findings suggest that metformin has potential for modulating microglia-mediated HIV-1 Tat neurotoxicity in HAND.

## Data Availability

The data that support the findings of this study are available on request from the corresponding authors, upon reasonable request.
